# Compression of time in double-step saccades

**DOI:** 10.1152/jn.00117.2024

**Published:** 2024-05-29

**Authors:** Eckart Zimmermann

**Affiliations:** Institute for Experimental Psychology, Heinrich Heine University Düsseldorf, Düsseldorf, Germany

**Keywords:** double-step saccade, saccades, time compression

## Abstract

Temporal intervals appear compressed at the time of saccades. Here, I asked if saccadic compression of time is related to motor planning or to saccade execution. To dissociate saccade motor planning from its execution, I used the double-step paradigm, in which subjects have to perform two horizontal saccades successively. At various times around the saccade sequence, I presented two large horizontal bars, which marked an interval lasting 100 ms. After 700 ms, a second temporal interval was presented, varying in duration across trials. Subjects were required to judge which interval appeared shorter. I found that during the first saccades in the double-step paradigm, temporal intervals were compressed. Maximum temporal compression coincided with saccade onset. Around the time of the second saccade, I found temporal compression as well, however, the time of maximum compression preceded saccade onset by about 70 ms. I compared the magnitude and time of temporal compression between double-step saccades and amplitude-matched single saccades, which I measured separately. Although I found no difference in time compression magnitude, the time when maximum compression occurred differed significantly. I conclude that the temporal shift of time compression in double-step saccades demonstrates the influence of saccade motor planning on time perception.

**NEW & NOTEWORTHY** Visually defined temporal intervals appear compressed at the time of saccades. Here, I tested time perception during double-step saccades dissociating saccade planning from execution. Although around the time of the first saccade, peak compression was found at saccade onset, compression around the time of the second saccade peaked 70 ms before saccade onset. The results suggest that saccade motor planning influences time perception.

## Graphical abstract

**Figure:**
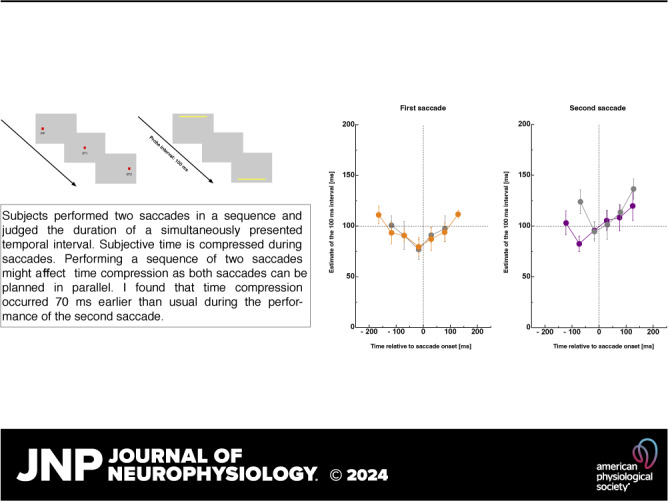

## INTRODUCTION

To maximize the efficiency with which we scan our visual environment, we perform saccades that are fast displacements of the eye ball. The increase in efficiency comes at a cost, which poses three challenges to the sensorimotor system: first, the high-speed shift of the eye moves the visual scene across the retina, thus producing a blurred motion stimulus. The term “saccadic omission” describes that in real life vision we do not perceive the intrasaccadic motion. Second, the visual system receives stable sensory input only before and after the saccade, but the received snapshots relate to different parts of the scene. A mechanism must update the internal representation of space for the vector of the saccade. Third, the disruption of the perceptual stream by the intrasaccadic period might also affect how we relate events in time. Since the visual motion is omitted during saccade execution, a temporal gap has been created in the otherwise regular visual input stream. If the saccade duration is not taken into account by neural temporal processing of visual input, event timing should be disturbed after every execution of a saccade.

Much research has addressed the first two problems (1, 2). Saccadic omission might result either from an active (3), from a passive mechanism (4), or from a storage of sensorimotor contingencies (5). Spatial updating might be yielded by receptive field shifts in the intraparietal cortex that remap the internal representation of space before execution of the saccade (2, 6). Regarding the third problem, it has been found that time appears compressed around execution of a saccade (7). Perisaccadic compression had previously been found for the perception of objects in space (8). When stimuli are presented close in time to saccade initiation, subjects mislocalize them to the position of the saccade target (8, 9). This shift in apparent position is bidirectional, resembling a compression of visual space. Likewise, temporal intervals appear to last much shorter when presented around the time of saccade execution (7). Even reversals in temporal order judgments could be observed, such that a stimulus presented in the intrasaccadic interval appears to have occurred earlier in the temporal stream than it actually was.

All three phenomena, suppression, compression of space, and compression of time share a similar time course (8). They start around 50–100 ms before saccade execution, peak around the time of saccade initiation, and decline while the saccade is in flight. It has been suggested that the similar time course is indicative of a common mechanism in the generation of the three phenomena (8). Since they are so tightly coupled to the performance of a saccade, their functional role might be to compensate for the three problems related to saccade execution, as mentioned earlier. We have shown in a previous study that comparable compression effects might also be found during ocular fixation (10). Temporal intervals appeared compressed when they were shown across the brief presentation of a whole field pattern mask that mimics perisaccadic vision. Saccadic time compression might thus result from the changes in vision that are related to saccade execution.

A method to isolate saccade execution factors from saccade planning is the double-step task (11, 12). Two saccade targets are presented in immediate succession to which subjects have to perform saccades sequentially. Each of the two targets is presented only briefly (e.g., 50 ms). Unlike in transsaccadic target displacement paradigms in which the saccade target is shifted to a new position while the eye is on flight (13), both targets are presented and extinguished before the saccade begins. Several pieces of evidence suggest that the two saccades in the double-step paradigm are planned in parallel. The duration of the intersaccadic interval can be as short as 20 ms (14), and saccade parameters like latency and duration covary with the total amplitude of the saccade sequence, rather than depending on the individual amplitudes (15). Planning the second saccade in the double-step paradigm can potentially start as soon as the second saccade target has been presented (16–23). The longer it takes to finish the execution of the first saccade, the more time is available to plan the second saccade and in consequence, the duration of the intersaccadic interval between the first and the second saccade could be shorter. Researchers found a negative correlation between the time to finish the first saccade and the duration of the intersaccadic interval, consistent with the idea of preplanning (14, 18). An electrophysiological study, recording in the frontal eye field (FEF), found that presaccadic activity of FEF movement neurons for the second saccade can be activated while the first is still underway (24).

We have previously shown that compression of space and transsaccadic updating are modulated in magnitude during the performance of the second saccade in the double-step task. The strength of saccadic compression of space (25, 26) and of transsaccadic updating (27) was reduced during the execution of the second but not during the first saccade. We interpreted these modulations as evidence that the entire saccade sequence is preprogrammed before execution of the first saccade. If saccadic suppression, saccadic compression of space, and transsaccadic updating are driven by saccade command signals and saccades programming occurs only before execution of the saccade sequence but not at the time of the second saccade, absence of these effects during the second saccade should be the consequence. Here, I wondered how time perception would be affected by the performance of the saccade sequence in the double-step task. Theoretical considerations and empirical evidence suggest that time perception might rely on motor structures (28, 29). If saccadic compression of time is motor-related it should be reduced during the second saccade in the double-step task like the effects mentioned earlier. Saccade remapping might be responsible for time compression. About 50 ms before saccade execution, receptive fields of intraparietal neurons shift to the location of the postsaccadic eye position (6), remapping the internal visual representation of external space. These receptive field shifts might interrupt the continuous processing of temporal visual information and thus lead to time compression. Alternatively, changes in stimulus visibility, which is around the time of saccade performance, might induce time compression.

## METHODS

### Participants

Five subjects (3 females, mean age: 32 yr) participated in all experiments. All subjects had normal or corrected-to-normal vision and participated with informed consent. Experimental procedures were approved by the local ethics committee of the psychological department of the Heinrich-Heine-University Düsseldorf. Written informed consent was obtained prior to the experiment in accordance with the declaration of Helsinki.

### Apparatus

Subjects were seated 45 cm from a Eizo FlexScan T57S with the head stabilized by a forehead rest. The visible screen diagonal was 20 in., resulting in a visual field of 40° × 30°. Stimuli were presented on the monitor with a vertical frequency of 120 Hz on a homogeneous gray background. Eye movements were monitored by the EyeLink 1000 system (SR Research), which samples gaze positions with a frequency of 1,000 Hz. Viewing was binocular, but only the dominant eye was recorded. A standard nine-point calibration was performed at the beginning of each block of trials. The system detected the start and the end of a saccade when eye velocity exceeded or fell below 22°/s and acceleration was above or below 4,000°/s^2^ respectively. All experiments were carried out in a complete dark environment. To avoid visibility of the screen borders, the display was covered with a transparent foil that reduced the luminance by about 2 log units.

### Double-Step Saccades

**Figure 1.**
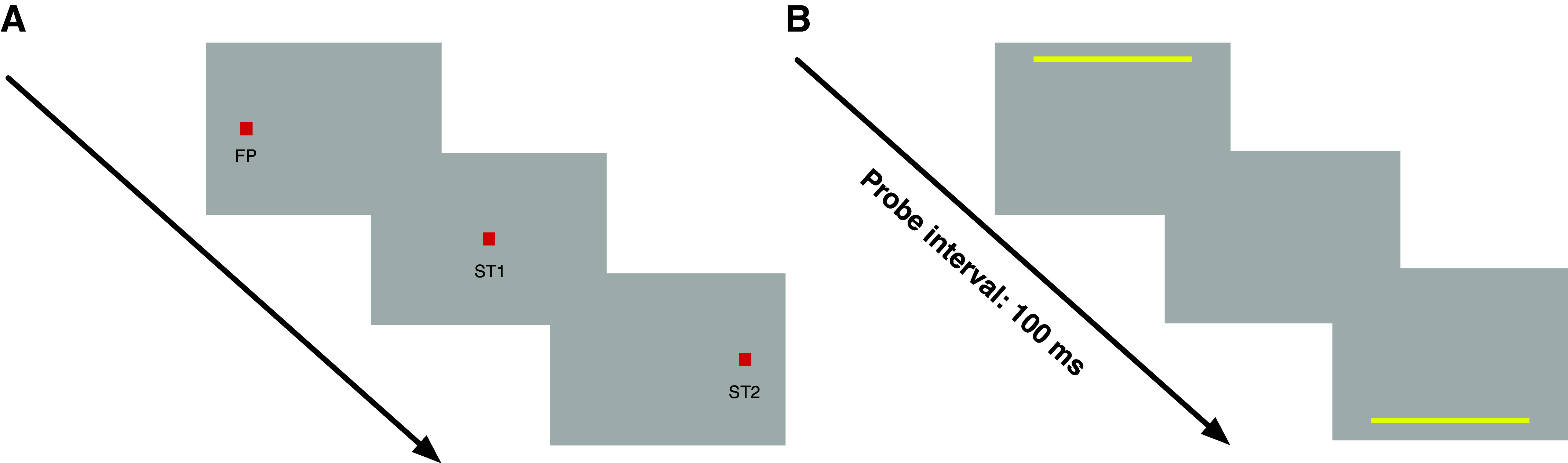
*A*: schematic illustration of the double-step saccade trials. At trial, subjects had to fixate on the fixation square (FP). A saccade target (ST1) was presented in screen center, requiring subjects to perform a 15° saccade. After 50 ms, ST1 disappeared and a second saccade target (ST2) was shown for 50 ms, 15° to the right of ST1. *B*: schematic illustration of the temporal interval stimuli. A bar was shown in the top of the screen for 8 ms. After 100 ms another bar was shown in the bottom of the screen for 8 ms. Subjects had to judge the duration of this interval against another pair of bars presented 700 ms later. The duration for the second bar pair varied between 8 and 200 ms.

A trial started with the presentation of a fixation square (1° × 1°) located 15° to the left of the screen center on the horizontal meridian (see Fig. 1*A*). Subjects were required to keep their gaze directed at the fixation point. After a randomly selected period between 1,250 and 1,750 ms, a saccade target (ST1) appeared in screen center for 50 ms. Simultaneously with the extinction of ST1, the second saccade target (ST2) was presented 15° to the right of the screen center on the horizontal meridian for 50 ms. Subjects were instructed to perform their saccade upon appearance of the first saccade target. At various time around execution of the saccades sequence, a pair of two bars was presented (see Fig. 1*B*). The first bar (red rectangle, size: 31.8° × 0.8°) was presented horizontally centered on the screen and 10° above the screen center for 8 ms. After 100 ms, a second bar was shown for 8 ms also horizontally centered but 10° below screen center. After 700 ms, another pair of bars was presented. The interval between the two bars in the second pair varied from trial to trial (between 8 and 200 ms in 7 steps, constant stimuli). Subjects had to judge which interval appeared shorter by pressing the left or right arrow key.

### Single-Step Saccades

The same subjects who performed the double-step sessions also performed the single-step sessions. After data in the double-step paradigm were collected, saccade amplitudes for the first and the second saccade were analyzed. In separate experiments, subjects were required to perform single saccades, whose amplitudes were matched either to the first saccade in the double-step paradigm or in a separate experiment matched to the second saccade. After a randomly selected period between 1,250 and 1,750 ms, a saccade target appeared in screen center for 50 ms to which subjects performed a saccade. A pair of bars was presented, the first bar in the top and the second in the bottom of the screen, separated by a temporal interval of 100 ms. Subjects had to compare the duration of the first interval against a second interval (varying between 8 and 200 ms in 7 steps, constant stimuli) presented 700 ms later. In sessions mimicking the first double-step saccade, the required saccade distance for each subject depended on the average saccade amplitude that subject performed in the first double-step saccade. Likewise, required saccade distances in sessions mimicking the second double-step saccade were determined by the average second saccade amplitude within each observer.

### Statistical Analysis

All trials were analyzed in which the primary saccade, either in the double-step or in the single-step sessions, had a minimum amplitude of 5°. Anticipatory saccades that started before saccade target onset were excluded from analysis.

Trials were sorted with respect of the temporal distance between the temporal center of the stimulus interval and saccade onset. Temporal estimates were binned into bins with a width of 50 ms. Within each bin, a psychometric function was determined by first averaging the responses within each subject for each comparison interval increment. A cumulative Gaussian function was fitted to these single-subject averaged data. The point of subjective equality was estimated by extracting the mean of the psychometric function for each observer in each bin.

For statistical analysis, either paired *t* tests were used or for factorial analyses, a nonparametric repeated-measures ANOVA was calculated, using the Aligned Rank Transform (30). Statistical significance was estimated with the Kenward–Roger approximation, a procedure that has been shown to result in acceptable Type 1 error rates even for smaller samples (31). Temporal estimates, i.e., the points of subjective equality, were tested for statistical significance in a 2 × 2 factorial design. The factor “saccade order,” included the first and the second saccade of the double-step paradigm and the factor “pre/peri,” included temporal estimates from the presaccadic and the perisaccadic range.

## RESULTS

I first analyzed the dynamics of both saccades in the double-step saccades. Subjects were required to perform two horizontal, rightward 15° saccades. On average, the first saccade had an amplitude of 16.00° (SE 1.09) and the second had an amplitude of 9.67° (SE 0.97). I asked the same subjects in a separate experiment to perform two single saccades, controlling for the effect of the first and the second double-step saccade. The required amplitude size of the single saccades was chosen for each individual participant such that the amplitude of the performed saccade would match the amplitudes of the average double-step saccades of that particular participant. Single saccades matched to the first saccades in the double-step paradigm had an average amplitude of 16.23° (SE 2.22) and single saccade matched to the second saccades had an average amplitude of 10.01° (SE 1.06). A nonparametric repeated-measures ANOVA revealed a significant main effect for the factor saccade order [*F*(1,4) = 51.61, *P* = 0.002]. Larger amplitudes in the first compared with the second saccade in the double-step paradigm have been reported in the literature and count as signature of preplanning of the entire saccade sequence (14).

**Figure 2.**
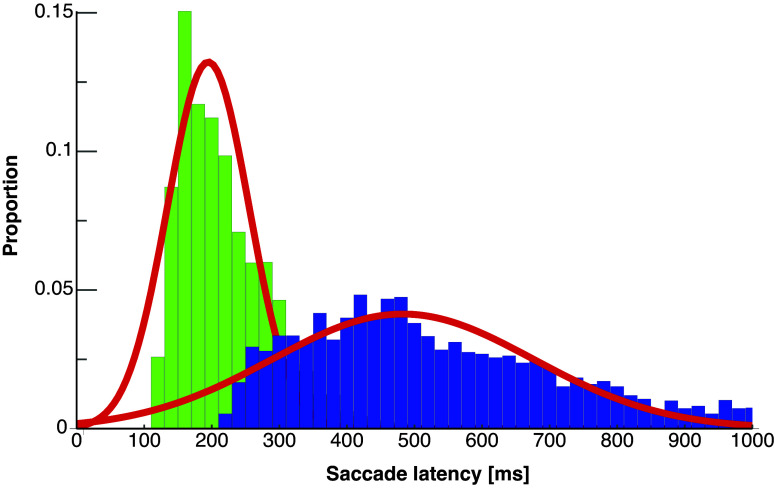
Distribution of saccade latencies in the double-step paradigm pooled across all participants. Latencies of the first saccades are shown in green and latencies of the second saccades are shown in blue. Red lines show Gaussian fits for latencies of the first (μ = 189.79, σ = 65.77) and the second saccades (μ = 470.42, σ = 206.77).

Subjects had to initiate their saccades as soon as the fixation point disappeared. Saccade latencies (see Fig. 2) for the first saccade were in the range usually observed in reactive saccades [189.56 ms (SE 25.37)]. The intersaccade interval defined as the duration between the end of the first saccade and the initiation of the second saccade was 236.32 ms (SE 39.69).

**Figure 3.**
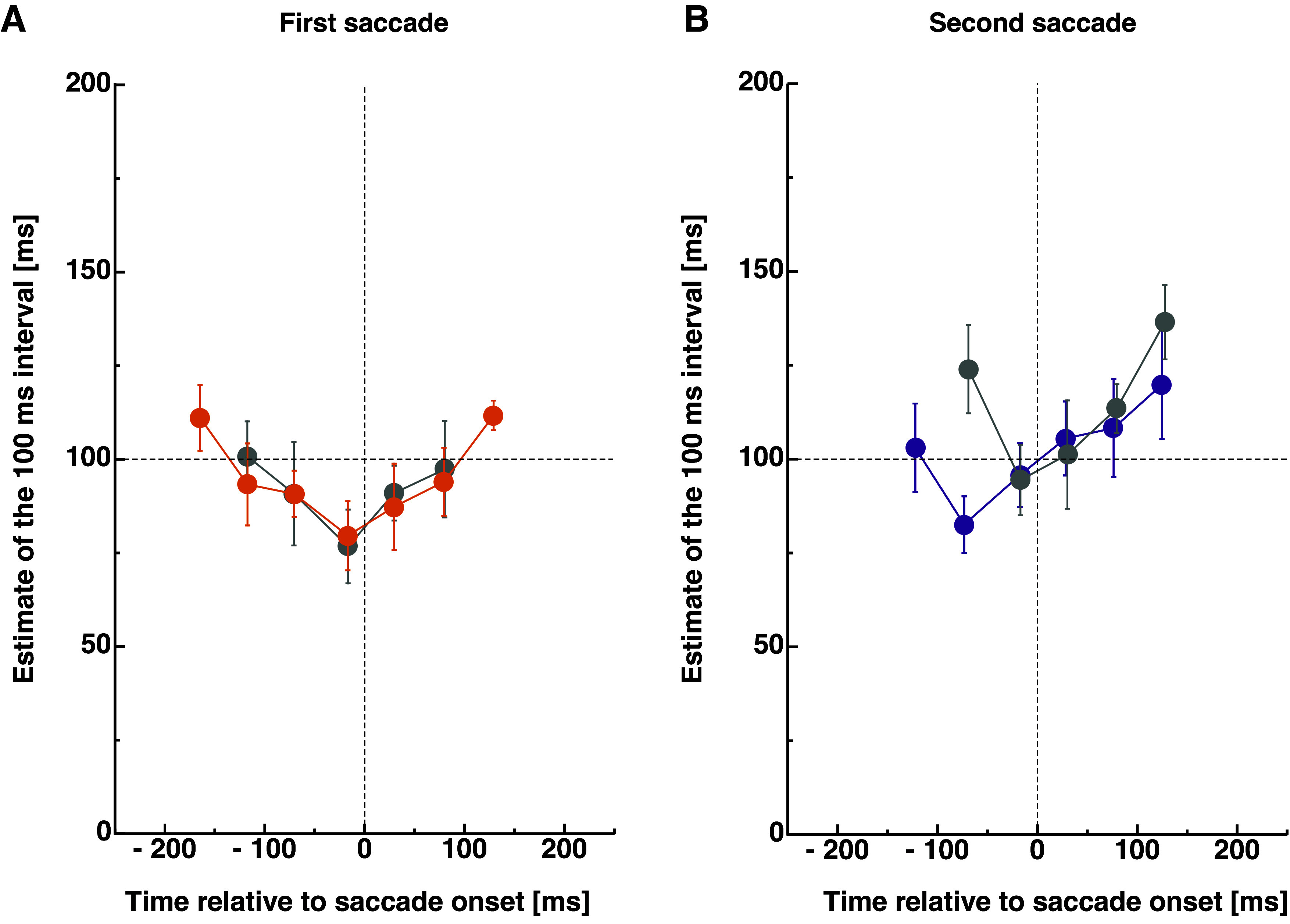
*A*: saccadic temporal compression curves for the first saccade in the double-step trials (shown in orange) and in amplitude-matched single saccades (shown in gray). Data were binned into bins with a width of 50 ms and sorted relative to saccade onset. Error bars represent SEM. *B*: saccadic temporal compression curves for the second saccade in the double-step trials (shown in purple) and in amplitude-matched single saccades (shown in gray). Error bars represent SEM.

I measured time perception during double-step saccades by presenting two briefly flashed horizontal bars at the top and the bottom of the screen that marked a probe interval of 100 ms. Across trials, the probe interval was presented at various times around the double-step saccade sequence. After an interstimulus interval of 700 ms, a second temporal interval was presented whose duration varied across trials. Subjects had to estimate which of both intervals appeared longer. I sorted data with respect to the temporal position of the interval center relative to saccade onset. I binned data into bins with a width of 50 ms. I determined points of subjective equality for each subject within each bin to estimate perceived duration. Figure 3 shows the time course of duration estimates averaged across observers. Data shown in color derive from the double-step saccades. Around −170 ms to saccade onset, subjects estimate the temporal interval to last close to 112.47 ms (SE 9.32). The closer the interval center is presented to saccade onset, the shorter the interval appears. About −16 ms to saccade onset, subjects judged the interval to last 80.97 ms (SE 9.59). A nonparametric repeated-measures ANOVA revealed a significant main effect for the factor “pre/peri” [*F*(1,4) = 22.53, *P* = 0.009].

Results for duration perception around the second saccade in the double-step paradigm are shown in Fig. 3*B* in purple. One can see that the underestimation of the temporal interval peaks much earlier before saccade onset than when measured in the first saccade. Duration estimates also increase again before the saccade has been initiated. To compare the saccade-induced temporal compression against time estimates during ocular fixation, I chose the time after execution of the second saccade, thus avoiding any potential influence of the intersaccadic interval. About 125 ms after saccade onset, subjects estimate the interval to last 108.27 ms (SE 13.04) and −73 ms prior to saccade onset, they estimate it to be 82.58 ms (SE 7.59). A nonparametric repeated-measures ANOVA revealed a significant main effect for the factor “pre/peri” [*F*(1,4) = 32.38, *P* = 0.005]. The magnitude of duration underestimation is similar in the amplitude-matched single saccades [at 127 ms: 136.57 (SE 9.92), at −17 ms: 94.43 (SE 9.39)]; however, the peak of underestimation is located much closer to saccade onset.

**Figure 4.**
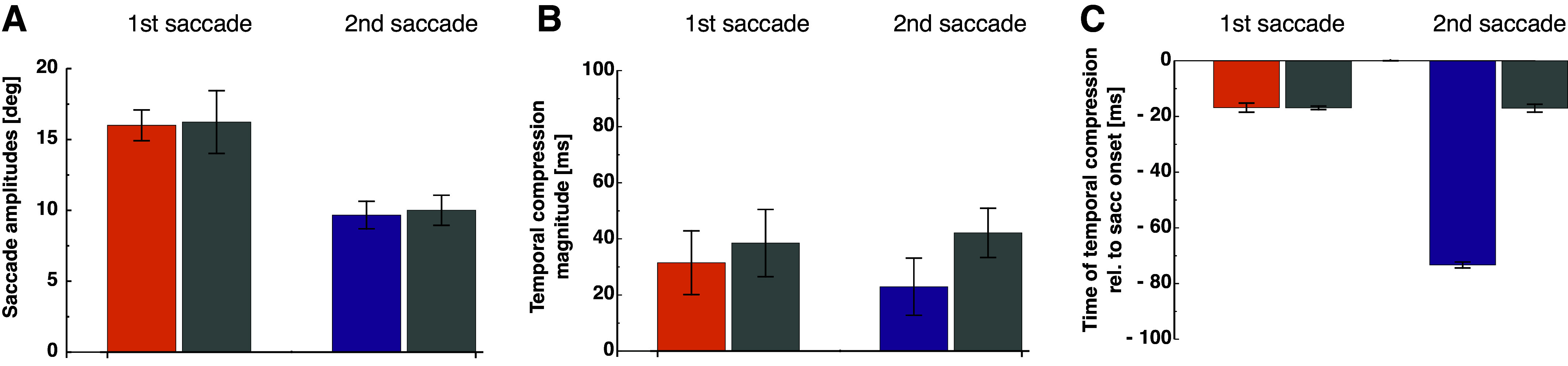
*A*: average saccade amplitudes in the double-step trials (shown in colors) and in the respective amplitude-matched single saccade. Error bars represent SEM. *B*: average temporal compression magnitude from bins with maximal temporal underestimation. Same conventions as in *A*. *C*: average time of temporal compression relative to saccade onset. Same conventions as in *A*.

Figure 4*C* shows average compression, defined as the difference in temporal estimates for the 100-ms interval at the peak of underestimation and the temporal estimates during ocular fixation. Comparable temporal compression magnitudes between 22 and 42 ms were found for all saccade types, which were statistically indistinguishable.

Average time of peak temporal compression relative to saccade onset is shown in Fig. 4*C*. A nonparametric repeated-measures ANOVA revealed a significant main effect for the factor saccade order [*F*(1,4) = 137.93, *P* < 0.001], a significant main effect for the factor paradigm [*F*(1,4) = 125.00, *P* < 0.001] and a significant interaction effect [*F*(1,4) = 90.91, *P* < 0.001].

## DISCUSSION

In this study, I investigated saccadic compression of time in a double-step task. Subjects performed two horizontal saccades in succession and judged the duration of a 100-ms interval that was presented at various times around execution of the saccade sequence. We found that subjects underestimated the temporal interval when its center coincided with the onset of the first saccade in the double-step task, thus demonstrating saccadic compression of time (7). However, around the time of the second saccade execution, the peak of time compression preceded saccade onset by about 70 ms.

When participants have to perform two saccades in the same direction, as in the double-step task, the first saccade is often larger than required—already deviating to the final target of the sequence—and the second saccade smaller (14). Any change in perception during performance of the second saccade could thus be due to the smaller saccade amplitude. The magnitude for some of the perceptual phenomena scales with saccade amplitude. For instance, saccade suppression (32) and saccade compression of space (33) have been shown to increase as a function of saccade amplitude size. After finishing the double-step task sessions, I asked the same subjects to perform single saccades. Their required size was chosen to respectively match the average amplitude of the first and the second saccade in the double-step task for each subject. Saccade time compression tested during amplitude-matched single saccades always coincided with saccade onset. Saccade amplitude sizes thus cannot explain the finding that the peak of time compression around the second saccade in the double-step paradigm occurs about 70 ms earlier than saccade onset.

For this reason, the results suggest that saccadic compression of time is related to saccade programming and not to saccade execution factors. The latter might modify time perception in several ways: first, attention shifting before saccade initiation could distort time estimates. Shortly before saccade execution, attention shifts to the saccade target (34–36). The change in attentional processing, which occurs at the same time as saccade time compression, could affect temporal processing. Distracting attention from the temporal estimation task leads to a decrease in perceived duration (37–45). Second, saccadic suppression, which decreases the sensitivity for perisaccadically presented stimuli (3) will affect the visibility of the interval markers. Visual contrast has been shown to slightly modify duration estimates (46). However, we have demonstrated in a previous study that saccade suppression during the first and the second saccade in the double-step paradigm remains unchanged when compared against amplitude-matched single saccades (27).

Since however these factors can be excluded, saccade programming might be responsible for saccadic time compression. In the first scenario, the mechanism updating visual space across the performance of saccades might be responsible for the temporal distortion. Under this interpretation, saccadic time compression resides in the linkage between visual and oculomotor areas. Several studies suggest that in the double-step task at least part of the programming of the second saccade is accomplished before execution of the first saccade (15–23). Visual receptive fields in the lateral intraparietal cortex shift to the location of the postsaccadic eye position shortly before saccade performance. If in the double-step task both saccades are preplanned in parallel, remapping might occur mostly at the time of the first saccade and covers the entire distance traveled by the two saccades. Consistent with this interpretation, we found in a previous study that perisaccadic compression (25, 26) occurs mostly at the time of the first saccade in the double-step paradigm. The data of the current study suggest that in the double-step paradigm as implemented, planning of the second saccade occurs about 70 ms ahead of its planning during single saccades. One might have expected planning of the second saccade to be fully in parallel to first saccade planning. However, this seems only to be the case in the rare cases in which intersaccade intervals last extremely short (14). The variable duration of the intersaccade intervals even within subjects suggests that second saccade planning in many trials has not finished after the end of the first saccade, since the most likely reason that the second saccade does not start is that its planning is still ongoing.

Temporal compression has been observed also during fixation when either the visibility of the flashed probe stimulus is reduced (47) or the stimulus is masked (10). It is yet unclear how the findings observed in fixation and those during saccades relate to each other. The explanation that stimulus visibility, which during saccades is reduced due to suppression (1), is responsible for the saccade temporal compression effects cannot hold for the current data since in the intersaccade interval visibility should be as high as before or after single saccades.

In conclusion, I found that executing a saccade motor plan interferes with visual time processing. The saccade time compression phenomenon cannot be caused by saccade execution factors, as time compression in the saccade double-step paradigm was only found during the first saccade. Likely explanations for time compression are either the occurrence of presaccadic remapping or the simultaneous usage of motor structures for saccades and for visual time estimates.

## DATA AVAILABILITY

Data will be made available upon reasonable request.

## GRANTS

This research was supported by the European Research Council under Project moreSense Grant Agreement Number 757184.

## DISCLOSURES

No conflicts of interest, financial or otherwise, are declared by the author.

## AUTHOR CONTRIBUTIONS

E.Z. conceived and designed research; performed experiments; analyzed data; interpreted results of experiments; prepared figures; drafted manuscript; edited and revised manuscript; approved final version of manuscript.
